# A Closer Look Into Brazil’s Healthcare System: What Can We Learn?

**DOI:** 10.7759/cureus.38390

**Published:** 2023-05-01

**Authors:** Alex Roman

**Affiliations:** 1 Neurological Surgery, Cleveland Clinic Abu Dhabi, Abu Dhabi, ARE

**Keywords:** healthcare quality, healthcare investment, population healthcare coverage, brazilian national health system, global healthcare system

## Abstract

Brazil’s healthcare system has made significant progress in recent years but still faces major challenges. In this editorial, we examine the greatest flaws and achievements of Brazil’s healthcare system, focusing on population coverage, quality metrics, spending over the last ten years, and estimates of per capita spending over the next decade. We discuss the role of the government, private sector, and civil society in shaping Brazil’s healthcare landscape and provide recommendations for improvement. Despite the challenges, Brazil has made impressive strides in healthcare, such as the implementation of the family health program, which has improved primary care access and reduced infant mortality rates. However, much work remains to be done, and Brazil must prioritize investment in healthcare infrastructure, workforce development, and the integration of digital technologies to ensure universal access to quality care for all.

## Editorial

Brazil's healthcare system is facing several challenges despite its vast territory and immense natural resources, which may prove the future potential to be further explored. The country has made significant progress in recent years to improve its healthcare system, but it still lags behind many other countries in terms of population coverage, quality metrics, and overall and efficient spending. This editorial examines the Brazilian healthcare system through the lens of global healthcare systems, focusing on its greatest flaws and achievements. Finally, we will offer recommendations for improving the Brazilian healthcare system.

The Brazilian healthcare system suffers from several significant flaws. Firstly, the issue of unequal access to healthcare services and resources, which is often limited in rural areas and regions with a high poverty rate, has been evidenced more difficult when in view of the vast territorial disparages [1]. This has resulted in significant disparities in healthcare outcomes between different regions of the country. Furthermore, low quality of care is an issue in Brazil, resulting in long wait times, a shortage of medical specialized and subspecialized professionals, and inadequate training and equipment for healthcare workers, contributing to poor health outcomes in some instances. Lastly, the Brazilian healthcare system suffers from a lack of coordination between different levels of care, resulting in fragmented care [2].

Despite these flaws, the Brazilian healthcare system has also achieved several significant successes. Perhaps the most notable is the creation of the Sistema Único de Saúde (SUS), a universal healthcare system that provides free healthcare to all Brazilian citizens. The SUS has been instrumental in improving access to healthcare services for many people, particularly those living in poverty. Another significant achievement of the Brazilian healthcare system is the reduction of infant mortality rates, decreasing from 53 deaths per 1,000 live births in 1990 to 12 deaths per 1,000 live births in 2019 [3]. This represents a significant improvement in health outcomes for women and children in Brazil. Moreover, the Brazilian healthcare system has made significant strides in the area of infectious disease control, such as HIV/AIDS, malaria, and tuberculosis, through prevention, treatment, and education initiatives. These infectious diseases’ treatments are completely covered by the national healthcare system (SUS), with significant advances in outcomes over the last decades in terms of overall survival and symptom-free survival.

In terms of population coverage, Brazil has made significant progress in recent years. The country has a universal healthcare system that theoretically covers all citizens, and the government has made significant investments in expanding access to healthcare services in rural areas and other underserved regions. However, when it comes to quality metrics, the Brazilian healthcare system still has ample room for improvement. The country ranks 125th out of 190 countries in the World Health Organization's ranking of healthcare systems, indicating significant challenges in ensuring that patients receive high-quality care, albeit the ranking system has been significantly challenged. In terms of spending, Brazil has increased its healthcare expenditure significantly over the last ten years although questioned in regard to efficient spending. Healthcare spending in Brazil increased from 8.3% of GDP in 2010 to 9.2% of GDP in 2018, but this still falls short of the average spending of other countries with similar levels of development [4].

The Brazilian government has set a target of spending 10% of GDP on healthcare by 2023. However, the COVID-19 pandemic has disrupted the country's economic and healthcare systems, and the government has diverted resources to the pandemic response. Nevertheless, projections suggest that per capita healthcare spending will increase from $848 in 2020 to $1,165 by 2030. The Brazilian government plans to improve healthcare infrastructure and expand access to healthcare services, particularly in underserved areas, to achieve this target [4].

To improve the Brazilian healthcare system, several recommendations could be implemented. Firstly, a comprehensive plan to address unequal access to healthcare services and resources is required, particularly in rural areas and regions with a high poverty rate. This will require significant investment in healthcare infrastructure and training of medical professionals, as well as the implementation of telemedicine and other technological solutions to improve access to healthcare services [5]. Secondly, the quality of care in the Brazilian healthcare system must be improved, with a focus on reducing waiting times (i.e., by better allocation of healthcare workers to areas of greater necessity, with personal and group incentives), improving the training of medical professionals, and ensuring that healthcare workers have access to the necessary equipment and resources to provide high-quality care.

Thirdly, the Brazilian healthcare system must be restructured to improve coordination between different levels of care, such as primary care, specialty care, and hospital care. This will require the universal and integrated implementation of electronic medical records and other digital solutions to ensure that patient information is accessible across different levels of care [5]. Finally, the Brazilian government must prioritize investments in preventive care, such as health promotion and disease prevention initiatives, to improve the overall health of the population and reduce the burden on the healthcare system.

The healthcare system in Brazil has indeed been studied from different perspectives. Belber et al. [6] provided a comprehensive overview of the current evidence on telehealth’s effectiveness in improving healthcare outcomes, enhancing patient satisfaction and reducing healthcare costs. Another study evaluated the effectiveness of primary healthcare in Brazil in the long run. Ferreira-Batista et al. (2023) [7] conducted a retrospective cohort study using data from Brazil's Family Health Strategy Program and found that primary healthcare reduced hospitalization rates and increased life expectancy. Another study by Baldissera et al. (2023) [8] identified the characteristics of work in primary care through the application of the SWOT matrix.

In the context of technology, Alves et al. (2021) [9] developed and validated a mobile application to promote self-care in adolescents with type 1 diabetes mellitus. The study showed that the application was effective in acquiring new knowledge and improving adherence to healthy practices. Similarly, Marcolino et al. (2021) [10] developed and implemented a decision support system to improve the control of hypertension and diabetes in a resource-constrained area in Brazil.

Finally, Dias et al. (2023) [11] studied the burden of rare diseases on caregivers in Latin America. The study found that caregivers faced several challenges in accessing healthcare services and experienced high levels of stress and social isolation. Soares et al. (2023) [12], in their study, focused on the implementation of a standardized handoff system in a tertiary care pediatric hospital in Brazil. The study showed that the implementation of the I-PASS system (a standardized handoff system) improved the quality of communication during handoffs and reduced medical errors. This study provides insights into strategies that could improve patient outcomes in tertiary care settings in Brazil.

In conclusion, the Brazilian healthcare system has achieved significant success in improving population coverage, reducing infant mortality rates, and controlling infectious diseases. However, the system still suffers from significant flaws, including unequal access to healthcare services, low quality of care, and a lack of coordination between different levels of care. To improve the Brazilian healthcare system, significant investments in healthcare infrastructure and training of medical professionals are required. The implementation of telemedicine and other digital solutions, universal and nationally integrated, as well as preventive care initiatives, should also be prioritized. By addressing these challenges, Brazil can achieve its goal of providing high-quality, accessible healthcare services to all of its citizens.
